# Enzymatic Primer-Extension with Glycerol-Nucleoside Triphosphates on DNA Templates

**DOI:** 10.1371/journal.pone.0004949

**Published:** 2009-03-23

**Authors:** Jesse J. Chen, Ching-Hsuan Tsai, Xin Cai, Allen T. Horhota, Larry W. McLaughlin, Jack W. Szostak

**Affiliations:** 1 Howard Hughes Medical Institute, and Department of Molecular Biology and Center for Computational and Integrative Biology, Massachusetts General Hospital, Boston, Massachusetts, United States of America; 2 Department of Chemistry, Merkert Chemistry Center, Boston College, Chestnut Hill, Massachusetts, United States of America; The Scripps Research Institute, United States of America

## Abstract

**Background:**

Glycerol nucleic acid (GNA) has an acyclic phosphoglycerol backbone repeat-unit, but forms stable duplexes based on Watson-Crick base-pairing. Because of its structural simplicity, GNA is of particular interest with respect to the possibility of evolving functional polymers by *in vitro* selection. Template-dependent GNA synthesis is essential to any GNA-based selection system.

**Principal Findings:**

In this study, we investigated the ability of various DNA polymerases to use glycerol-nucleoside triphosphates (gNTPs) as substrates for GNA synthesis on DNA templates. Therminator DNA polymerase catalyzes quantitative primer-extension by the incorporation of two glyceronucleotides, with much less efficient extension up to five glyceronucleotides. Steady-state kinetic experiments suggested that GNA synthesis by Therminator was affected by both decreased catalytic rates and weakened substrate binding, especially for pyrimidines. In an attempt to improve pyrimidine incorporation by providing additional stacking interactions, we synthesized two new gNTP analogs with 5-propynyl substituted pyrimidine nucleobases. This led to more efficient incorporation of gC, but not gT.

**Conclusions:**

We suggest that directed evolution of Therminator might lead to mutants with improved substrate binding and catalytic efficiency.

## Introduction

**Figure 1:**
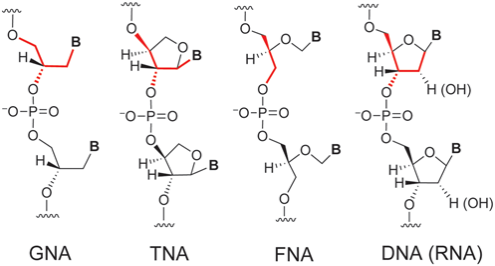
Structures of GNA, TNA, FNA, and DNA (RNA). The backbone similarity is highlighted in red.

The ribose and 2-deoxyribose components of the backbones of RNA and DNA have long been thought to play a critical role in stabilizing Watson-Crick base pairing and maintaining fidelity in polymerase-mediated information transfer. Having survived billions of years of evolution on Earth, ribose has been considered to be one of the indispensable building blocks of contemporary life [1]. However, recent studies from the Eschenmoser group of nucleic acid analogs with altered sugar-phosphate backbones demonstrate dramatically that D-ribofuranose is not a chemical requirement for the ability of a prebiotically plausible nucleic acid to function as the genetic information carrier [2]. Indeed, a nucleic acid with a simpler backbone structure, such as (3′→2′)-α-L-threose nucleic acid (TNA, Figure 1), might have been an evolutionary progenitor of DNA or RNA [3].

Recently, the Meggers group synthesized *S*-Glycerol nucleic acid (GNA), a nucleic acid analog with an acyclic, phosphoglycerol repeating unit (Figure 1) [4], [5]. Like TNA, GNA is a nucleic acid with a simplified backbone that can form stable duplexes. As a first step toward understanding the evolutionary potential of GNA, we have recently shown that Bst DNA polymerase can catalyze faithful (but limited) DNA synthesis on a GNA template even though the DNA product cannot form a stable duplex with the GNA template [6]. This surprising observation suggests that, in contrast to common belief [7], stable heteroduplex formation is not required for genetic information transfer between two nucleic acid systems—a short base-paired region stabilized by the enzyme appears to be sufficient.

**Figure 2:**
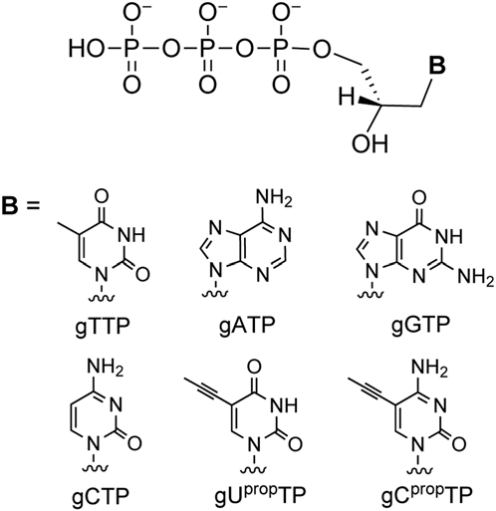
Structures of glyceronucleoside triphosphates (gNTPs).

In addition to exploring GNA molecules as a genetic information carrier, we are also interested in probing their ligand binding and catalytic abilities. We envision that an *in vitro* selection strategy, similar to that used in RNA aptamer or artificial ribozyme selection [8], can be used to isolate functional GNA constructs. This selection strategy requires a polymerase that can synthesize GNA in a template-directed fashion. We have previously synthesized the four canonical glycerol-nucleoside triphosphates (gNTPs with A, G, T, or C, Figure 2) and demonstrated that several DNA polymerases can incorporate a single glyceronucleotide onto a DNA primer/template using gNTPs as the substrates [9]. Incorporation of a second glyceronucleotide was not observed in that study, which we attributed to the poorly constrained nature of the acyclic glycerophosphate backbone of GNA and/or the unstable nature of a GNA/DNA duplex [9].

In the present study, we show that certain DNA polymerases can incorporate at least two GNA residues onto a growing primer. Kinetic analysis of Therminator-catalyzed single glyceronucleotide incorporation suggested that pyrimidine glyceronucleotides were weakly bound substrates. In an attempt to improve pyrimidine incorporation, we synthesized two 5-propynyl substituted gNTP analogs, to improve the base-stacking interaction between the incoming gNTP and the terminal nucleotide of the primer (Figure 2). This modification led to improved efficiency of incorporation of gCTP, but not of gTTP.

## Results

### Polymerase Screens

**Figure 3:**
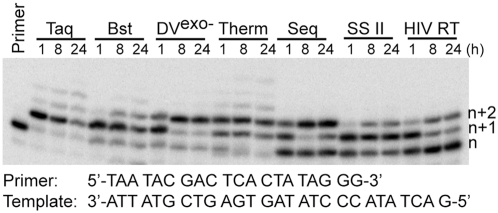
Primer extension analysis of GNA synthesis mediated by polymerases.

Using a primer extension assay, we screened a number of polymerases (including Taq, Bst, DeepVent (exo-), Therminator, Sequenase, Superscript II, and HIV-1 reverse transcriptase) for their ability to catalyze GNA synthesis on a DNA template. In order to enhance our chances of observing primer-extension by more than just one base as previously observed [9], we used long incubations under a variety of conditions. We found that a number of polymerases were able to incorporate more than one GNA monomer at a 1∶1 enzyme∶primer/template ratio (Figure 3). The Therminator polymerase utilized most of the primer and generated a significant fraction of +2 extended products within 1 h (Figure 3). After 8 h, Deep Vent exo- polymerase had a higher yield of +2 extended primer, but Therminator appeared to synthesize small amounts of +3, +4, and +5 products. We therefore decided to characterize primer-extension and substrate utilization by Therminator in greater detail. Raising the assay temperature from 55°C to 75°C (Therminator's optimum temperature) resulted in decreased efficiency of GNA incorporation. Therefore, all the subsequent kinetic studies were performed at 55°C. Neither the addition of Mn^2+^ nor substitution of adenosine with diaminopurine-2′-deoxyriboside in the template significantly improved the efficiency of GNA synthesis by this enzyme, using the four canonical gNTPs.

The products of Therminator-catalyzed, single and double glyceronucleotide incorporation were characterized by MALDI-TOF MS. The expected mass difference between a primer extended with one deoxyribonucleotide vs. one glyceronucleotide is 42 Daltons. As shown in Table 1, the observed masses for single glyceronucleotide or deoxyribonucleotide incorporation on Template A are consistent with the calculated values. The identity of the products of two sequential nucleotide incorporations (gTMP+dAMP, gTMP+gAMP, 2 gAMP, or 2 gGMP on Template B) was also confirmed by MALDI-TOF MS (Table 1).

**Table 1:** MALDI-TOF MS analysis of single and double nucleotide incorporation by Therminator DNA polymerase a.

	XY	NTP substrate	Nucleotide incorporated	Calcd	Obsd
Template A	CA	none	none	6126.1	6126.2
	AG	TTP	T	6430.3	6431.5
	TG	dATP	dA	6439.3	6439.5
	CA	dGTP	dG	6455.3	6455.3
	GA	dCTP	dC	6415.3	6415.3
	AG	gTTP	gT	6388.3	6388.3
	AG	gU^prop^TP	gU^prop^	6412.3	6411.9
	TG	gATP	gA	6397.3	6396.8
	CA	gGTP	gG	6413.3	6413.4
	GA	gCTP	gC	6373.3	6373.3
	GA	gC^prop^TP	gC^prop^	6411.3	6410.8
Template B	AT	gTTP+dATP	gT+dA	6701.5	6700.0
	AT	gTTP+gATP	gT+gA	6659.5	6658.9
	TT	gATP	gA+gA	6668.5	6667.7
	CC	gGTP	gG+gG	6700.5	6699.8

a Sequences of the primer and the template:

Primer 5′-TAA TAC GAC TCA CTA TAGGG-3′.

Template : 3′-ATT ATG CTG AGT GAT ATC CC **XY** ACA TCT ATC-5′.

Template B: 3′-ATT ATG CTG AGT GAT ATC CC **XY** GCA TCT ATC-5′.

### Kinetic analysis of glycerol-nucleotide addition to a DNA primer

To obtain insight into the process of GNA synthesis catalyzed by Therminator on DNA templates, we performed steady-state kinetic experiments and measured the *k*_cat_ and K_m_ values for the four canonical gNTP substrates (Figure 2). The kinetic parameters for addition of a single glyceronucleotide to an all-DNA primer are summarized in Table 2. Because the kinetic experiments were performed at 55°C instead of Therminator's optimum temperature of 75°C, decreased *k*_cat_ values were observed for regular dNTPs (2–7 s^−1^ at 55°C, Table 2) compared with those at 75°C (∼15 s^−1^ calculated from specific activity provided by the manufacturer). Therminator showed similar *k*_cat_ values for gATP compared to dATP, and for gGTP compared to dGTP, while slower kinetics were observed for gTTP and gCTP (by 5-fold and 3-fold, respectively) compared with TTP and dCTP. All 4 gNTPs showed higher K_m_ values than the corresponding dNTPs, with an ∼20 fold increase for gATP and gGTP and a 200–300 fold increase for gTTP and gCTP. As a result, the decrease of catalytic efficiency (*k*_cat_/K_m_) was much more dramatic for pyrimidine than for purine gNTPs (1000- fold versus only 20-fold). Such discrimination between purine and pyrimidine triphosphate substrates is not observed for threose-nucleoside triphosphates (tNTPs) [10]. One possible explanation for this result is that base-stacking interactions between the incoming nucleoside triphosphate and the primer play a more prominent role in gNTP binding than in dNTP or tNTP binding.

**Table 2:** Kinetic analysis of single-nucleotide incorporation by Therminator DNA polymerase a.

XY^b^	NTP	K_m_ (µM)	*k*_cat_ (s^−1^)	*k*_cat_/K_m_ (s^−1^ M^−1^)
TG	dATP	0.59±0.11	3.7±0.5	6.2×10^6^
CA	dGTP	0.47±0.27	2.0±0.3	4.3×10^6^
AG	TTP	0.67±0.14	4.2±0.4	6.4×10^6^
DG	TTP	0.35±0.10	6.8±0.6	1.9×10^7^
GA	dCTP	0.32±0.17	3.2±0.8	1.0×10^7^
TG	gATP	14.0±6.7	4.7±0.8	3.4×10^5^
CA	gGTP	10.6±4.7	2.3±0.1	2.1×10^5^
AG	gTTP	129.4±77.3	0.8±0.3	5.8×10^3^
DG	gTTP	54.3±8.3	1.6±0.4	3.0×10^4^
AG	gU^prop^TP	139.5±14.5	1.5±0.3	1.1×10^4^
DG	gU^prop^TP	41.4±4.7	1.7±0.2	4.1×10^4^
GA	gCTP	89.5±38.6	1.3±0.6	1.4×10^4^
GA	gC^prop^TP	3.3±0.4	1.9±0.2	5.8×10^5^

a Sequences of the primer and the template:

Primer: 5′-TAA TAC GAC TCA CTA TAGGG-3′.

Template: 3′-ATT ATG CTG AGT GAT ATC CC **XY** ACA TCT ATC-5′.

b D denotes 2,6-diaminopurine-2′-deoxyribonucleotide.

### Synthesis of 5-propynyl substituted glycerol-nucleoside triphosphates

**Figure 4:**
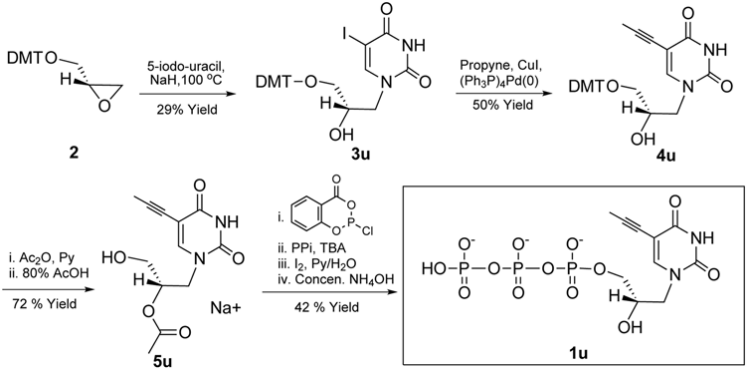
Synthesis of 5-propynyluridine glyceronucleoside triphosphate (1u).

**Figure 5:**
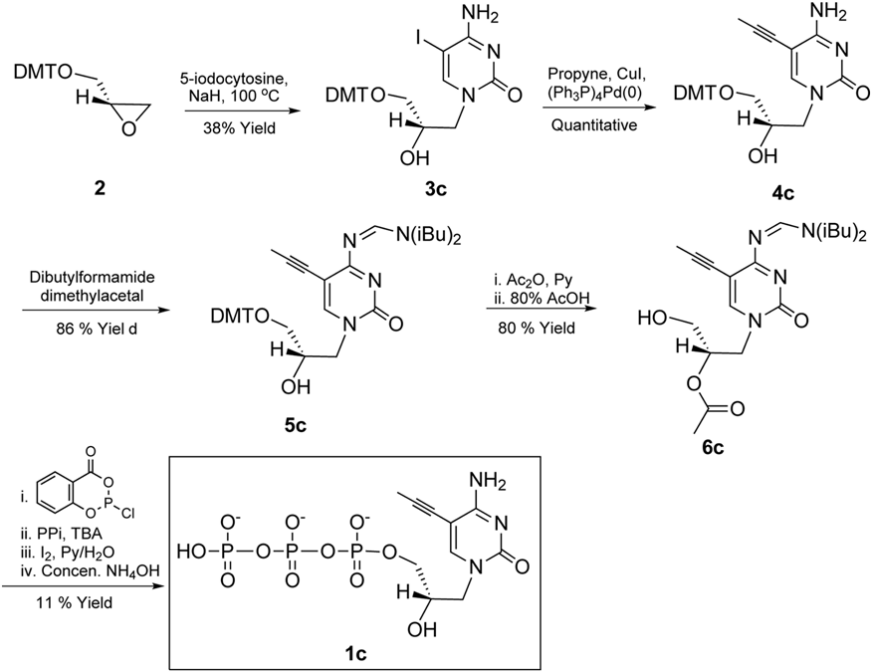
Synthesis of 5-propynylcytidine glyceronucleoside triphosphate (1c).

In order to test the hypothesis that poor enzymatic incorporation of pyrimidine gNTPs reflects poor base-stacking, we synthesized the C-5-propynyl substituted pyrimidine nucleoside triphosphates (Figure 2). This co-planar nucleobase modification has been proposed to increase base stacking and hydrophobic interactions between base pairs [11]. In the case of GNA synthesis, we thought that a 5-propynyl group in pyrimidine gNTPs might improve binding to the primer/template complex in the active site of polymerase. The synthetic schemes for the preparation of 5-propynyl substituted gNTPs (gU^prop^TP and gC^prop^TP, or **1u** and **1c**) are shown in Figures 4 and 5. Two key intermediates, the 5-propynyl pyrimidine glycerol-nucleosides (**4u** and **5c**), were prepared from 5-iodo-substituted precursors (**3u** and **3c**) and propyne by Sonogashira coupling (Figure 4 and 5) [12], [13]. A di-butylaminomethylidene group was used to protect the exocyclic amine of **3c** instead of an acetyl or benzoyl group in order to avoid a potential cyclization side-reaction involving the amide and the 5-propynyl group [14]. The presence of a propynyl group in **4u** and **5c** was confirmed by the characteristic chemical shift (4–5 ppm) of the methyl carbon in ^13^C NMR [15] together with ^1^H-NMR and ESI-MS analysis. gU^prop^TP and gC^prop^TP (**1u** and **1c**) were synthesized from the corresponding nucleosides (**5u** and **6c**) using the one-pot, salicylchlorophosphorin approach developed by Ludwig and Eckstein [16]. The final purified products **1u** and **1c** were characterized by ^1^H- and ^31^P-NMR and by ESI-MS. In addition, **1u** and **1c** have similar UV absorption profiles to those reported for 5-propynyl-deoxyribonucleosides [15].

### Kinetics of DNA primer-extension using 5-propynyl gNTPs

We measured K_m_ and *k*_cat_ for Therminator-catalyzed DNA primer-extension, using 5-propynyl gUTP and gCTP as substrates. The results suggest that a stronger base stacking interaction does increase the catalytic efficiency of gNTP incorporation, although the effect is more prominent for gC^prop^TP than for gU^prop^TP (Figure 2). Compared to gCTP, gC^prop^TP had a much lower K_m_ (3.3 µM vs. 89.5 µM) and a slightly faster *k*_cat_ (1.9 s^−1^ vs. 1.3 s^−1^) (Table 2). As a result, the catalytic efficiency (*k*_cat_/K_m_) of Therminator with gC^prop^TP was 40 fold higher than that with gCTP. In fact, gC^prop^TP had the lowest K_m_ and highest *k*_cat_/K_m_ of all 6 gNTPs. In contrast, the effect of 5-propynyl substitution on gUTP was modest, and gU^prop^TP showed a similar K_m_ value to gTTP with an ∼2 fold increase in *k*_cat_ (Table 2). When diaminopurine was used in the template, both gTTP and gU^prop^TP had a significantly lower K_m_ (54.3 µM and 41.4 µM, respectively) than those measured with the dA-containing template (129.4 µM and 139.5 µM, respectively) with slightly increased *k*_cat_ (Table 2). These results suggest that both H-bonding and base stacking contribute to binding of gNTPs to the primer/template complex and that the synergy of these two interactions determines the overall catalytic efficiency.

### Kinetic analysis of dNMP incorporation on a GNA-terminated primer

We synthesized a primer containing a single GNA monomer (gT) at the 3′-terminus in order to study the effect of the positioning of the nucleophile (2′-OH of GNA vs. 3′-OH of DNA) on the catalytic efficiency of Therminator polymerase. However, kinetic parameters for a 3′-gT-terminated primer were more difficult to obtain than with an all-DNA primer. Under steady-state conditions with a low enzyme∶primer/template ratio, we were not able to observe significant primer-extension even at saturating gNTP concentrations. At a 1∶1 enzyme∶primer/template ratio (1 µM), ∼15% extension of the 3′-gT-terminated primer was observed after 24 h with gATP and gGTP, but not with gTTP and gCTP. This observation suggests that, in the active site of Therminator, the conformation of the terminal GNA nucleotide, especially its 2′-OH group, is not optimal (or is poorly constrained) for nucleophilic attack on the incoming triphosphate. The catalytic efficiency is further reduced by the low affinity of Therminator for gNTP substrates, especially in the case of gTTP and gCTP.

To quantitatively assess the effect of a 3′-terminal gT residue on the catalytic efficiency of continued polymerization, we determined the kinetic parameters for the incorporation of a single deoxyribonucleotide by Therminator, using a 3′-gT primer. Compared with the all-DNA primer (Table 2), the 3′-gT primer caused 2–4 fold decrease in *k*_cat_ (Table 3). In addition, an increase in K_m_ was also observed, which again was more prominent for pyrimidines (∼80 fold) than for purines (4–8 fold) (Table 3). These results suggest that the presence of gT at the 3′-terminus of the primer may interfere with essential conformational changes of the enzyme during its catalytic cycle, resulting in both slower *k*_cat_ and increased K_m_ values.

**Table 3:** Kinetic analysis of single deoxyribonucleotide incorporation by Therminator using a GNA-terminated primer a.

XY	NTP	K_m_ (µM)	*k*_cat_ (s^−1^)	*k*_cat_/K_m_ (s^−1^ M^−1^)
TG	dATP	4.7±2.0	0.7±0.2	1.4×10^5^
CA	dGTP	2.1±1.5	1.0±0.1	4.7×10^5^
AG	TTP	50.7±38.5	0.9±0.2	1.8×10^4^
GA	dCTP	21.4±7.6	1.7±0.6	7.8×10^4^

a Sequences of the primer and the template (the lower case denotes the GNA sequence):

Primer: 5′-TAA TAC GAC TCA CTA TAG GG t.

Template: 3′-ATT ATG CTG AGT GAT ATC CC A **XY** CAT CTA TC-5′.

## Discussion

Previous studies have shown that certain polymerases are capable of template-directed primer-extension by using as substrates nucleotide analogs with either a shortened (TNA) or an acyclic (flexible nucleic acid, or FNA, Figure 1) sugar moiety [10], [17], [18]. The active sites of some polymerases are clearly flexible enough to accommodate major sugar modifications [19], [20]. However, compared with TNA or FNA, GNA contains both an acyclic and a shortened repeating unit (Figure 1), and it is therefore not surprising that the enzymatic polymerization of gNTPs is quite difficult. In this study, we screened a series of polymerases and identified Therminator as the most efficient GNA polymerase. Not surprisingly, Therminator was also the most efficient polymerase identified in during previous efforts to synthesize TNA or FNA on DNA templates [10], [18].

Our kinetic analysis of primer-extension with gNTP substrates has provided some insight into the particular problems that prevent efficient Therminator-catalyzed GNA synthesis. Analysis of the addition of the first glycerol-nucleotide to a DNA primer revealed that all six gNTPs examined (the four canonical nucleobases plus the two propynyl-pyrimidines) had *k*_cat_ values that were similar to those of the corresponding natural dNTPs. Decreased catalytic efficiency was mainly due to higher K_m_ values, and thus is most likely due to weaker gNTP binding. The same observation was made previously for TNA synthesis catalyzed by Therminator [10]. These results suggest that altered sugar structure on an incoming NTP does not prevent Therminator from efficiently orienting the triphosphate moiety of the NTP for nucleophilic attack by the 3′-hydroxyl of the DNA primer. The major kinetic difference between gNTPs and threose-nucleoside triphosphates (tNTPs) is in the incorporation of additional nucleotides to the growing primer, where the attacking nucleophile becomes the 2′-OH. In the case of TNA, which has a relatively rigid threose ring, it appears that Therminator can effectively position the 2′-OH for nucleophilic attack. As a result, for both tNTP and dNTP incorporation, the *k*_cat_ values for a TNA-terminated primer are similar to those obtained with an all-DNA primer [10]. However, with a gT-terminated primer, the efficiency of gNTP incorporation dropped dramatically (Table 3). These results indicate that the main obstacle to continued GNA synthesis is a poorly positioned (or poorly constrained) 2′-OH group at the end of a GNA primer.

Previous studies suggest that a 5-propynyl group on a pyrimidine nucleoside triphosphate is well tolerated by a variety of DNA polymerases [21]–[23]. For example, Kuwahara *et al.* showed that 5-propynyl dUTP and dCTP were efficiently utilized by several thermophilic polymerases in PCR with high accuracy [24]. However, there are no systematic studies on the effect of the 5-propynyl group on the kinetics of polymerase-mediated (d)NTP incorporation. Wang and Shaw showed that for MMLV reverse transcriptase, 5-propynyl-dUTP had a similar K_m_ (14.1 µM) and a slightly faster *k*_cat_ (0.023 s^−1^) compared with TTP (14.4 µM and 0.015 s^−1^) [23]. Our results show that the 5-propynyl substitution can significantly improve gCTP incorporation by Therminator, but has surprisingly little effect on gTTP incorporation. It appears that the stronger H-bonding of the G:C base-pair acts synergistically with the favorable base stacking interaction induced by the 5-propynyl group. Other effects, such as perturbation of pKa of N4 of cytosine, might also contribute to a better binding of gC^prop^TP compared to gCTP. As for gTTP and gU^prop^TP, our results show that the stronger base-pairing interaction with diaminopurine in the template did improve the substrate binding and overall efficiency. However, the 5-propynyl group did not significantly increase the catalytic efficiency of gTMP incorporation in either adenosine- or diaminopurine-containing templates. This observation is consistent with the previous studies by Seela *et al.*, who showed that the duplex stabilizing effect caused by the 5-propynyl group (measured by increase of Tm) was smaller for dT (0.75–1.5°C) than for dC (2–3°C)[25]. Our kinetic analysis suggests that the increased base-stacking interaction caused by the 5-propynyl group in gU^prop^TP might be offset by a weakening of the U^prop^:A base pairing interaction relative to T:A, due to the perturbed basicity of N3 in 5-propynyl-uracil (p*K*_a_ = 8.7) compared with that in thymine (p*K*_a_ = 9.8) (F. Seela, Personal Communication).

In addition to DNA templates, we also attempted to identify polymerases that can use GNA templates. Unfortunately, none of the polymerases we studied showed significant activity with gNTP substrates and GNA templates, even when the primer and primer-binding region were composed of DNA. This lack of activity is not surprising given the challenges of binding and pre-organizing a GNA template in the active site, and binding and orienting the gNTP substrate for catalysis. Natural DNA or RNA polymerases have their active sites optimized for binding B-form or A-form duplexes. Recent X-ray structural studies suggest that the conformation of a GNA duplex differs significantly from either B-form or A-form, although the GNA duplex studied also contained a metallo-base-pair [26]. It is likely that significant engineering of the enzyme active site would be required to evolve an efficient GNA-dependent GNA polymerase using the existing Therminator polymerase as a starting point.

## Materials and Methods

### Materials

Reagents and solvents were purchased from Sigma-Aldrich. DNA oligonucleotides were purchased from IDT and were purified by denaturing polyacrylamide gel electrophoresis. Dibutylformamide dimethylacetal [14], *R*-3-*O*-(4,4′-dimethoxytrityl)-glycidol (**2**) [27] , and gNTPs (A, G, T, C) [9] were prepared according to published procedures. Taq DNA polymerase was purchased from Roche (1 U is defined as the amount of enzyme that incorporates 10 nmol of total dNTPs into acid precipitable DNA within 30 min at 75°C). Therminator, Deep Vent (exo-), and Bst polymerases were purchased from New England Biolabs (1 U is defined as the amount of enzyme that incorporates 10 nmol of total dNTPs into acid insoluble material in 30 min at 75°C for Therminator and Deep Vent (exo-) or at 65°C for Bst). Sequenase was purchased from USB (1 U is defined as the amount of enzyme that catalyzes the incorporation of 1 nmol of nucleotide into acid insoluble form in 30 s at 37°C). Superscript II reverse transcriptase was purchased from Invitrogen (1 U is defined as the amount of enzyme that incorporates 1 nmol of deoxyribonucleotide into acid-precipitable material in 10 min at 37°C using poly(A)oligo(T)_12­18_ as template/primer). HIV-1 reverse transcriptase was purchased from Worthington (1 U is defined as the amount of enzyme that incorporates 1 nmol of tritiated TMP into acid precipitable products using poly(A)/oligo(dT)_12–18_ as the template/primer in 20 min at 37°C, pH 8.3). Thermostable inorganic pyrophosphatase was purchased from New England Biolabs (1 U is defined as the amount of enzyme that generates 1 µmol of phosphate per minute from inorganic pyrophosphate at 75°C in 50 mM Tricine, 1 mM MgCl_2_, 0.32 mM PPi, pH 8.5). Flash column chromatography was performed using silica gel from Sigma-Aldrich (Grade 9385, 230–400 mesh) with solvents indicated below. ^1^H-, ^31^P-, and ^13^C-NMR experiments were performed on a Varian 400 MHz spectrometer. Chemical shifts are reported in ppm with reference to TMS (0.00 ppm) for ^1^H, phosphoric acid (0.00 ppm) for ^31^P, or CDCl_3_ (77.16 ppm) for ^13^C. Coupling constants are reported in Hz. Low-resolution mass spectrometry (LSMS) analysis was performed on a Bruker Daltonics Esquire 6000 mass spectrometer. All reactions were performed under a dry argon atmosphere.

### Synthesis of S-1-(5-iodouracil-1-yl)-3-O-(4,4′-dimethoxytrityl)-2,3-propanediol (3u)

5-iodouracil (3.6 g, 15 mmol) and sodium hydride (125 mg in 60% mineral oil, 3.0 mmol) were suspended in 25 mL of anhydrous dimethylformamide and the reaction mixture was stirred at room temperature for 30 min. *R*-3-*O*-(4,4′-dimethoxytrityl)-glycidol (**2**) (15 mmol) in 25 mL of anhydrous dimethylformamide was added to the reaction mixture drop-wise over 5 min. The reaction mixture was then heated to 100°C and stirred for 1.5 h. The solvent was then evaporated in vacuo and the resulting pellet was extracted with 50 mL of CH_2_Cl_2_ twice. The organic layer was combined, washed twice with saturated sodium bicarbonate (100 mL×2) and once with brine (100 mL), dried over Na_2_SO_4_, and evaporated in vacuo. Compound **3u** was further purified by silica gel chromatography (0.5%–1% MeOH/CH_2_Cl_2_) to afford **3u** as amorphous light yellow solid (2.7 g, 4.4 mmol, 29%). ^1^H NMR (400 MHz, CDCl_3_) δ: 7.72 (1H, s), 7.41(1H, s), 7.39(1H, s), 7.26–7.32 (6H, m), 6.84(4H, d, J = 6.8), 4.04–4.07(2H, m), 3.78(6H, s), 3.65 (1H, dd, J = 8.4, 14.8), 3.18(2H, m); ^13^C NMR (100.5 MHz, CDCl_3_) δ: 160.6, 158.8, 151.1, 150.5, 144.5, 135.6, 130.1, 128.2, 128.0, 127.2, 113.4, 86.7, 69.2, 67.3, 64.5, 55.4, 52.0; LRMS calcd for [M+Na]^+^ (C_28_H_27_IN_2_NaO_6_): 637.0811, obsd, 637.0.

### Synthesis of S-1-(5-propynyl-uracil-1-yl)-3-O-(4,4′-dimethoxytrityl)-2,3-propanediol (4u)

Compound **3u** (1.87 g, 3.05 mmol) was dissolved in 20 mL of anhydrous dimethylformamide. Triethylamine (0.81 mL, 6.0 mmol), CuI (116 mg, 0.61 mmol), and tetrakis(triphenylphosphine)palladium(0) (352 mg, 0.31 mmol) were added to the reaction mixture at room temperature. Propyne gas was then bubbled through the reaction mixture at room temperature for 4 h. The solvent was then evaporated in vacuo and the resulting pellet was extracted twice with CH_2_Cl_2_ (50 mL×2). The organic layer was combined, washed twice with saturated sodium bicarbonate (100 mL×2) and once with brine (100 mL), dried over Na_2_SO_4_, and evaporated in vacuo. Compound **4u** was further purified by silica gel chromatography (0.5% MeOH/CH_2_Cl_2_) to afford the purified product as light yellow foam (0.8 g, 1.5 mmol, 50%). ^1^H NMR (400 MHz, CDCl_3_) δ: 8.94 (1H, br), 7.46 (1H, s), 7.42(1H, s), 7.40(1H, s), 7.23–7.31 (6H, m), 6.83(4H, d, J = 7.2), 4.04–4.08(2H, m), 3.79(6H, s), 3.61 (1H, dd, J = 8.4, 14.4), 3.16(2H, m), 3.04 (1H, br), 2.02(3H, s); ^13^C NMR (100.5 MHz, CDCl_3_) δ: 162.5, 158.7, 150.5, 148.1, 144.6, 135.6, 130.1, 128.1, 128.0, 127.1, 113.4, 100.1, 90.9, 86.6, 70.1, 69.1, 64.6, 55.4, 52.1, 4.8; LRMS calcd for [M+Na]^+^ (C_31_H_30_N_2_NaO_6_): 549.2002, obsd, 549.1.

### Synthesis of S-1-(5-propynyl-uracil-1-yl)-2-O-acetyl-2,3-propanediol (5u)

Compound **4u** (0.2 g, 0.38 mmol) was mixed with 4 mL of 10% acetic anhydride in anhydrous pyridine. The reaction mixture was stirred at room temperature for 15 min and was evaporated in vacuo. The crude product was dissolved in 50 mL CH_2_Cl_2_, washed twice with saturated sodium bicarbonate (50 mL×2) and once with brine (50 mL), dried over Na_2_SO_4_, and evaporated in vacuo. The pellet was then treated with 5 mL of 80% acetic acid in H_2_O at room temperature for 30 min. The mixture was then evaporated and the crude **5u** was purified by silica gel chromatography (2.5% MeOH/CH_2_Cl_2_) to afford **5u** as white foam (72 mg, 0.27 mmol, 72%). ^1^H NMR (400 MHz, CDCl_3_) δ: 9.61 (1H, br), 7.42 (1H, s), 5.10 (1H, m), 4.17 (1H, dd, J = 4.4, 14.4), 3.88 (1H, dd, J = 6.4, 14.4), 3.73 (2H, m), 2.11 (3H, s), 2.04 (3H, s). ^13^C NMR (100.5 MHz, CDCl_3_) δ: 170.5, 162.6, 150.7, 146.8, 101.1, 91.4, 71.9, 69.8, 60.9, 48.6, 21.1, 4.8. LRMS calcd for [M+Na]^+^ (C_12_H_14_N_2_NaO_5_): 289.0800, obsd, 289.0.

### Synthesis of S-1-(5-propynyl-uracil-1-yl)-2-O-acetyl-3-O-triphospho-2,3-propanediol (1u)

Compound **5u** (53 mg, 200 µmol) was rendered anhydrous by evaporation with anhydrous pyridine (1 mL×3) and was dissolved in 0.8 mL of pyridine/dioxane (1∶3). To this mixture, 220 µL of 1 M 2-chloro-4*H*-1,3,2-benzodioxaphosphorin-4-one in dioxane was added and the reaction mixture was stirred at room temperature for 10 min. A solution (0.6 mL) of 0.5 M tetrabutylammonium pyrophosphate in dimethylformamide was then added and the reaction mixture was stirred for another 10 min. A solution (7.5 mL) of 1% iodine in pyridine/H_2_O (98∶2) was then added to the above mixture. After 15 min, 5% Na_2_SO_3_ aqueous solution was added dropwise until the reaction mixture turned from dark brown to light yellow. The final mixture was evaporated in vacuo and treated with concentrated ammonia solution (10 mL) overnight at room temperature. The mixture was then evaporated in vacuo and the crude product **1u** was purified by preparative RP-HPLC using a Varian Microsorb 100-8 C18 column (250×21.4 mm) with a linear gradient from 0 to 20% acetonitrile in 5 mM triethylammonium acetate (pH 7.0) over 20 min with a flow rate of 15.0 mL/min. The fraction containing **1u** (retention time 4.5–5.5 min) was combined and lyophilized to yield 84.4 µmol of **1u** (42% yield) based on the UV absorption (ε_291 nm_: 11.3 mM^−1^ cm^−1^). ^1^H NMR (400 MHz, D_2_O) δ: 7.84 (1H, s), 4.04 (3H, m), 4.02 (1H, m), 3.80 (1H, dd, J = 6.2, 14.0), 2.00 (3H, s). ^31^P NMR (161.8 MHz, D_2_O) δ: −6.3 (d, J = 19.4), −9.90 (d, J = 19.6), −21.7 (t, J = 19.6). LRMS calcd for [M-H]^−^ (C_10_H_14_N_2_O_13_P_3_): 462.9714, obsd, 463.0.

### Synthesis of S-1-(5-iodocytosine-1-yl)-3-O-(4,4′-dimethoxytrityl)-2,3-propanediol (3c)

5-iodocytosine (2.37 g, 10.0 mmol) and potassium carbonate (276 mg, 2.0 mmol) were suspended in 20 mL of anhydrous dimethylformamide and the mixture was stirred at room temperature for 20 min. *R*-3-(4,4′-dimethoxytrityl)-glycidol **(2)** (15 mmol) in 20 mL of anhydrous dimethylformamide was added to the reaction mixture drop-wise over 5 min at room temperature. The reaction mixture was then stirred at 80°C for 16 h. The solvent was evaporated in vacuo and the resulting pellet was extracted with 50 mL of CH_2_Cl_2_ twice. The organic layer was combined, washed twice with saturated sodium bicarbonate (100 mL×2) and once with brine (100 mL), dried over Na_2_SO_4_, and evaporated in vacuo. Compound **3c** was further purified by silica gel chromatography (1%–3% methanol/CH_2_Cl_2_) as light yellow solid (2.3 g, 3.8 mmol, 38%). ^1^H NMR (400 MHz, CDCl_3_) δ: 7.63 (1H, s), 7.48 (1H, br), 7.42(1H, s), 7.40(1H, s), 7.20–7.32 (6H, m), 6.83(4H, d, J = 6.8), 5.50 (1H, br), 4.14–4.25 (2H, m), 3.79(6H, s), 3.75 (1H, m), 3.23 (1H, dd, J = 10, 4.8), 3.00 (1H, dd, J = 10, 6.4); ^13^C NMR (100.5 MHz, CDCl_3_) δ: 164.2, 158.7, 157.1, 152.8, 144.8, 135.9, 135.8, 130.0, 128.1, 128.0, 127.0, 113.4, 86.5, 69.3, 64.6, 55.9, 55.4; LRMS calcd for [M+Na]^+^ (C_28_H_28_IN_3_NaO_5_): 636.0971, obsd, 635.9.

### Synthesis of S-1-(5-propynylcytosine-1-yl)-3-O-(4,4′-dimethoxytrityl)-2,3-propanediol (4c)

Compound **3c** (1.3 g, 2.0 mmol) was dissolved in 15 mL of anhydrous dimethylformamide. Triethylamine (0.54 mL, 4 mmol), CuI (76 mg, 0.4 mmol), and tetrakis(triphenylphosphine)palladium(0) (231 mg, 0.2 mmol) were added to the solution at room temperature. Propyne gas was then bubbled through the reaction mixture for 4 h with stirring at room temperature. The solvent was then evaporated in vacuo and the resulting pellet was extracted with 50 mL of CH_2_Cl_2_ twice. The organic layer was combined, washed twice with saturated sodium bicarbonate (100 mL×2) and once with brine (100 mL), dried over Na_2_SO_4_, and evaporated in vacuo. Compound **4c** was further purified by silica gel chromatography (2% methanol/CH_2_Cl_2_) to yield amorphous light yellow solid (1.1 g, 2.1 mmol, quantitative yield). ^1^H NMR (400 MHz, CDCl_3_) δ: 7.74 (1H, br), 7.40–7.42 (3H, m), 7.23–7.31 (6H, m), 6.82(4H, d, J = 6.8), 5.75 (1H, br), 4.19–4.24 (2H, m), 4.11 (1H, m), 3.77 (6H, s), 3.74(1H, dd, J = 14, 6.8), 3.24 (1H, dd, J = 9.6, 5.2), 2.97 (1H, dd, J = 9.6, 6.8), 2.02 (3H, s); ^13^C NMR (100.5 MHz, CDCl_3_) δ: 165.5, 158.6, 156.9, 149.1, 144.8, 135.9, 135.8, 130.0, 128.1, 128.0, 127.0, 113.3, 92.5, 91.9, 86.5, 70.2, 69.8, 94.3, 55.3, 4.6; LRMS calcd for [M+H]^+^ (C_31_H_32_N_3_O_5_): 526.2342, obsd, 526.2.

### Synthesis of S-1-(5-propynyl-4-N-dibutylaminomethylidene-cytosine-1-yl)-3-O-(4,4′-dimethoxytrityl)-2,3-propanediol (5c)

Compound **4c** (0.34 g, 0.65 mmol) was dissolved in 7.5 mL of methanol. Dibutylformamide dimethylacetal (0.73 mL, 3.2 mmol) was added drop-wise at room temperature. The reaction mixture was stirred at room temperature for 3 h and was then evaporated in vacuo to afford the crude product as yellow oil. Compound **5c** was further purified by silica gel chromatography (1–2% methanol/CH_2_Cl_2_) as white foam (0.37 g, 0.56 mmol, 86%). ^1^H NMR (400 MHz, CDCl_3_) δ: 8.78 (1H, s), 7.54 (1H,s), 7.42 (2H, d, J = 8.4), 7.20–7.32 (7H, m), 6.83 (4H, d, J = 6.8), 4.30 (1H, d, J = 4.8), 4.24 (1H, dd, J = 2.8, 14.0), 4.08 (1H, m), 3.84 (1H, dd, J = 6.8, 14.0), 3.78 (6H, s), 3.61 (2H, m), 3.34 (2H, t, J = 7.2), 3.27 (1H, dd, J = 5.2, 9.6), 2.97 (1H, dd, J = 6.8, 9.6), 2.02 (3H, s), 1.58–1.69 (4H, m), 1.30–1.41 (4H, m), 0.98 (3H, t, J = 7.2), 0.94 (3H, t, J = 7.6); ^13^C NMR (100.5 MHz, CDCl_3_) δ: 171.4, 162.8, 158.6, 158.3, 157.5, 150.1, 144.9, 136.0, 135.8, 130.0, 128.03, 127.99, 126.9, 113.3, 100.3, 89.2, 86.5, 72.6, 70.1, 64.4, 55.3, 54.8, 52.5, 46.1, 31.1, 29.0, 20.2, 19.9, 13.8, 4.6; LRMS calcd for [M+H]^+^ (C_40_H_49_N_4_O_5_): 665.3703, obsd, 665.3.

### Synthesis of S-1-(5-propynyl-4-N-dibutylaminomethylidene-cytosine-1-yl)-2-O-acetyl-2,3-propanediol (6c)

The procedure to synthesize **6c** is similar to that of **5u**. The yield of **6c** from 0.2 g of **5c** (0.3 mmol) was 0.11 g (0.24 mmol, 80%). ^1^H NMR (400 MHz, CDCl_3_) δ: 8.83 (1H, s), 7.50 (1H, s), 5.03 (1H, m), 4.21 (1H, dd, J = 4, 14.4), 4.07 (1H, dd, J = 5.2, 14.4), 3.63 (1H, dd, J = 4, 12.4), 3.53 (1H, dd, J = 6.4, 12.4), 3.47 (2H, m), 3.18 (2H, d, J = 7.6), 2.11 (3H, s), 2.05 (3H,s), 0.95 (6H, d, J = 6.4), 0.93 (6H, d, J = 6.8); ^13^C NMR (100.5 MHz, CDCl_3_) δ: 171.5, 170.2, 159.1, 157.0, 148.8, 101.5, 89.8, 72.7, 72.3, 60.8, 59.5, 54.3, 48.5, 27.5, 26.6, 21.1, 20.4, 20.0, 4.6. LRMS calcd for [M+H]^+^ (C_21_H_33_N_4_O_4_): 405.2496, obsd, 405.1.

### Synthesis of S-1-(5-propynylcytosine-1-yl)-3-O-triphospho-2,3-propanediol (1c)

The procedure to synthesize **1c** is similar to that of **1u**. The crude product of **1c** after concentrated ammonia deprotection was first purified by anion-exchange liquid chromatography using DEAE A25 resin with a gradient of 0–0.5 M triethylammonium bicarbonate. The fraction containing **1c** was combined, evaporated in vacuo, and desalted using the preparative RP-HPLC as described for **1u** (retention time 8.5 min). The final yield of **1c** was 22.3 µmol (11%) based on the UV absorption (ε_295 nm_: 7.7 mM^−1^ cm^−1^). ^1^H NMR (400 MHz, D_2_O) δ: 7.85 (1H, s), 4.10 (2H, m), 4.01 (1H, m), 3.77 (1H, dd, J = 10, 14.4), 3.52 (1H, dd, J = 7.6, 14.4), 2.02 (3H, s). ^31^P NMR (161.8 MHz, D_2_O) δ: −5.34 (d, J = 21.5), −9.75 (d, J = 19.1), −21.5 (t, J = 21.1). LRMS calcd for [M-H]^−^ (C_10_H_15_N_3_O_12_P_3_): 461.9874, obsd, 462.0.

### Enzyme Screen

A DNA primer (5′-TAA TAC GAC TCA CTA TAG GG-3′) was 5′-labeled with ^32^P and annealed to a DNA template (3′-ATT ATG CTG AGT GAT ATC CCA TAT CAG-5′, the underlined denotes the region base-paired with the primer). The primer extension experiments of GNA synthesis on the DNA template were preformed with 50 nM primer/template, four gNTPs (A, G, T, and C, 100 µM each), 1 U of Thermostable pyrophosphatase, and polymerase (Taq, 2.5 U; Therminator, 1 U; Deep Vent (exo-), 1 U; Bst, 4 U, Sequenase, 6.5 U; Superscript II, 100 U; HIV-1 reverse transcriptase, 5 U) in a final volume of 10 µL containing appropriate buffers supplied by the manufactures. The reaction mixtures were incubated at 55°C for thermophilic enzymes or 37°C for mesophilic enzymes. At 1, 8, and 24 h, an aliquot of 2 µL was removed from the reaction mixture and was analyzed by 20% denaturing polyacrylamide gel electrophoresis.

### MALDI-TOF Mass Spectrometry

The single-nucleotide extension reaction mixture (50 µL) contained 20 µM pre-annealed primer/template, 250 µM appropriate dNTP or gNTP, 1 U of thermostable pyrophosphatase, and 1 U of Therminator DNA polymerase. The reactions mixtures were incubated at 55°C for 5 minutes for dNTPs or 8 h for gNTPs. The reaction mixtures were then precipitated by adding 200 µL of ethanol, 50 µL of 2 M ammonium acetate, and 0.5 µL of 3 mg/mL glycogen solution. The samples were redissolved in 0.2 M triethlyammonium acetate buffer (pH 7.0) and were absorbed on C18 Zip Tips (Millipore). Samples were eluted with 1.5 µL of a matrix solution containing a 2∶1 mixture of 52.5 mg/mL 3-hydroxypicolinic acid in 50% acetontrile and 0.1 M ammonium citrate in water. Eluents were directly spotted onto a stainless steel MALDI-TOF plate and were analyzed in positive mode on a Voyager MALDI-TOF mass spectrometer (Applied Biosystems) with an average of 200 scans.

### Steady-State Kinetics

Kinetic measurements were performed as previously described using 5′-[^32^P]-labeled primer and the appropriate template for each dNTP or gNTP [10], [28]. The single nucleotide extension reactions were initiated by mixing 10 µL of 2× dNTP or gNTP solution with 10 µL of a solution containing the remaining reaction components. The final reaction mixture of 20 µL contained 1 µM primer/template, 0.05–10 µM dNTP or 5–500 µM gNTP, 0.5 U of thermostable pyrophosphatase, and 8.2 nM Therminator in 1× Thermopol buffer (20 mM Tris-HCl, 10 mM KCl, 10 mM (NH_4_)_2_SO_4_, 20 mM MgSO_4_, 0.1% Triton X-100, 100 µM DTT, pH 8.8). The reaction mixtures were incubated at 55°C. At each time point, an aliquot of 4 µL was removed from the mixture and was quenched by mixing with 4 µL of solution containing 8 M urea, 100 mM EDTA, and 0.05% xylene cyanol and bromophenol blue. Single nucleotide extension was analyzed by 20% denaturing polyacrylamide gel electrophoresis and quantified using a PhosphorImager (Molecular Dynamics) as described before [10].
